# Case Report of the Unusual Presentation of Stridor in an Elderly Patient Following a Cervical Fracture

**DOI:** 10.21980/J8V926

**Published:** 2020-01-15

**Authors:** Benjamin Travers, Rachel Dearden, Shanna Jones, Michael Opsommer

**Affiliations:** * Oakland University William Beaumont School of Medicine, Rochester, MI; ^ Beaumont Health System, Department of Emergency Medicine, Troy, MI

## Abstract

This report describes a unique presentation of stridor in an elderly patient who presented to the emergency department (ED) with neck and back pain after a fall down a flight of stairs. The patient had no airway issues on initial evaluation in the resuscitation room, but developed stridor and respiratory distress after returning from imaging. The unexpected stridor and respiratory distress were alleviated with basic airway maneuvers that included airway repositioning and a breathing treatment. Cervical computed tomography (CT) showed a cervical fracture and a retropharyngeal hematoma, which was responsible for the partial obstruction of this patient’s upper airway leading to delayed stridor. Following management of the respiratory distress, the patient was diagnosed with and treated for central cord syndrome. The key takeaway from this case is that there should be a low level of suspicion for impending upper airway compromise in elderly patients presenting to the ED with a cervical spine injury.

**Topics:**

Stridor, retropharyngeal hematoma, cervical spine fracture, central cord syndrome.

[Fig f1-jetem-5-1-v15] [Fig f2-jetem-5-1-v15]

**Figure f1-jetem-5-1-v15:**
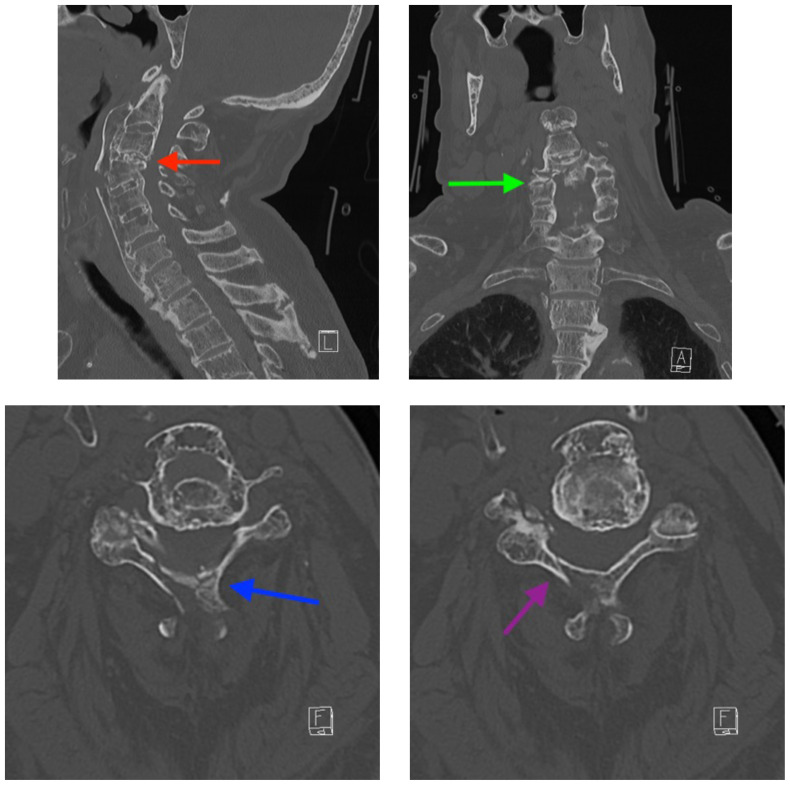


**Figure f2-jetem-5-1-v15:**
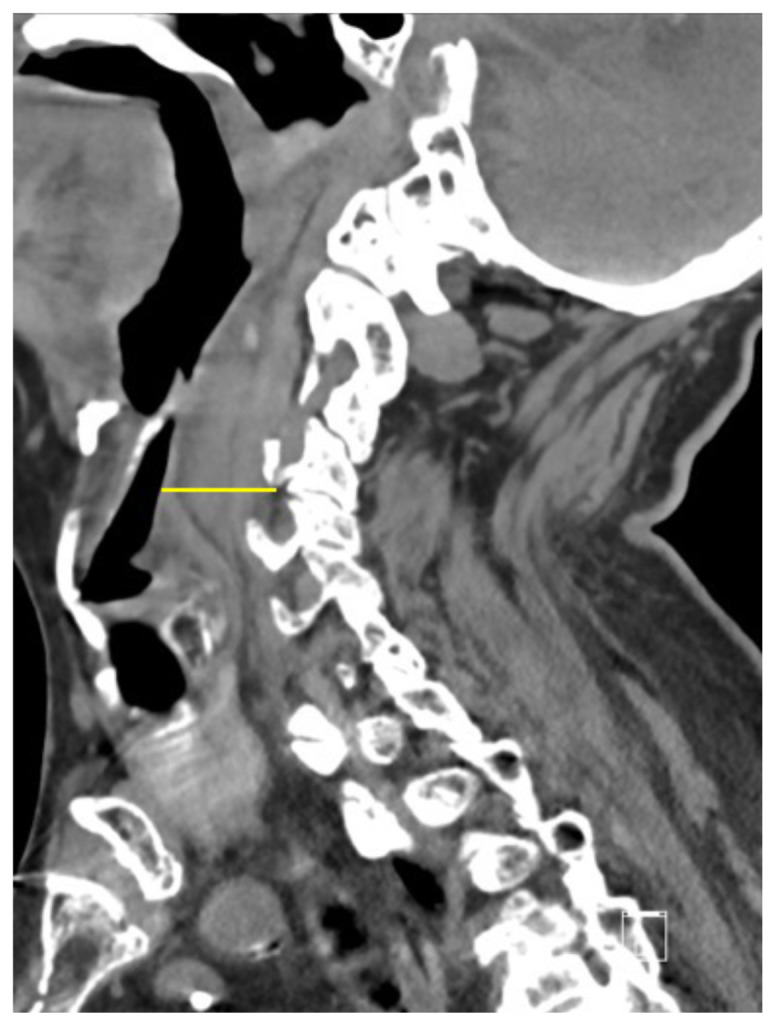


## Introduction

An elderly patient presented to the ED with neck and back pain after a fall down a flight of stairs and was diagnosed with a cervical spine fracture. The patient later developed stridor and moderate respiratory distress while in the ED, which was unexpected based on the initial evaluation. Review of the CT showed a retropharyngeal hematoma, which can be seen in the setting of trauma to the cervical spine.^1^,^2^,^3^,^4^ A review of the literature demonstrated a limited number of case reports involving delayed stridor secondary to a cervical spine fracture and retropharyngeal hematoma.^1^

## History of present illness

A 75-year old female with a history of multiple myeloma presented to the ED with generalized neck and back pain after an unwitnessed fall down a flight of ten stairs. She was noted to have midline tenderness over the cervical spine and bilateral weakness in the upper extremities on physical exam, which was suggestive of central cord syndrome. Neurologic function was normal at baseline, and she was not taking any blood thinners. Given the traumatic mechanism of injury, a cervical collar was placed, and she was sent for a head and cervical spine CT to evaluate for an intracranial hemorrhage or spinal fracture. While awaiting results of the CTs, she developed stridor with moderate respiratory distress. She received a jaw thrust maneuver to open her airway and was started on a DuoNeb breathing treatment. These interventions alleviated the respiratory distress and no further airway management was required. The differential diagnosis was expanded to include spinal fracture, intracranial hemorrhage, central cord syndrome, and phrenic nerve compression secondary to a spinal cord injury.

## Significant findings

The cervical CT was significant for a transverse fracture through the C4 vertebral body (see red arrow), lateral facet (green arrow), spinous process (blue arrow), and right lamina (purple arrow) as well as surrounding edema and retropharyngeal thickening (yellow line), best appreciated on sagittal view.

## Patient course

The patient was admitted to the surgical intensive care unit and was diagnosed with central cord syndrome (CCS) and underlying diffuse idiopathic skeletal hyperostosis (DISH). She underwent anterior cervical open reduction – internal fixation (ORIF) of C3–C6 and posterior decompression cervical laminectomy C3–C6 fusion by orthopedic surgery. The patient did well postoperatively and was discharged to an extended care facility for rehabilitation.

## Discussion

The retropharyngeal space lies anterior to the cervical spinal cord and is clinically significant in the setting of trauma or infection.^1^,^2^,^3^,^4^ Cervical trauma can damage small vessels in the neck, which slowly bleed into the retropharyngeal space leading to a retropharyngeal hematoma.^1^,^2^,^3^ Airway compromise is the major complication of a retropharyngeal hematoma and often presents as delayed respiratory distress.^1^,^4^ In this case, the patient’s cervical fracture likely led to a small bleed that accumulated in the retropharyngeal space and eventually compressed the upper airway causing stridor. Additionally, the patient was noted to have underlying anterior DISH. This condition increased her vulnerability to cervical fracture and prevertebral swelling, as a hypercalcified cervical spine is unable to withstand the forces of trauma with the same elasticity as a healthy spine.^5^

The retropharyngeal hematoma itself is often self-limiting and resolves over 2–4 weeks, but the respiratory distress requires immediate intervention.^1^,^4^ Depending on the severity of the respiratory distress, it may be appropriate to start with repositioning the patient’s airway before moving on to more advanced airway procedures. 4,6 A jaw thrust maneuver is often effective because this technique can open up the airway and improve oxygen delivery to the lungs. 4,6 It may also be appropriate to start a breathing treatment because if blood oxygen saturation can be maintained above 90%, then intubation may not be needed.^4^ If basic airway maneuvers fail, a definitive airway such as intubation or a surgical airway may be required.^4^,^7^

Due to mechanical obstruction of the airway in the setting of a retropharyngeal hematoma, preparations for a difficult airway should also be made, with multiple types of airways and surgical airway tools available.^7^ The patient should be preoxygenated and the physician should carefully consider the use and choice of induction agents and neuromuscular blocking agents in these cases.^4^,^7^ For instance, the use of ketamine as an induction agent in the setting of respiratory distress would provide the additional benefit of bronchodilation.^8^ In the setting of trauma, succinylcholine is the ideal neuromuscular blocking agent for its fast-acting and short-duration effects, assuming the patient’s potassium levels are within the normal range.^9^ Following stabilization of the respiratory distress, the retropharyngeal hematoma may require surgical evacuation if it was initially large or did not resolve with conservative treatment after 2–4 weeks.^4^

Following management of the respiratory distress, this patient was diagnosed with and treated for a cervical facture and central cord syndrome (CCS). The classic presentation of CCS includes bilateral pain/temperature loss at the level(s) of injury and upper limb weakness, which were seen in this patient.^10^,^11^ The classic symptoms of CCS are explained by damage to the spinothalamic tract and lateral corticospinal tract in the central portion of the spinal cord.^10^,^11^ The spinothalamic tract carries pain/temperature sensations and decussates in the anterior white commissure, so damage to the anterior white commissure causes bilateral pain/temperature loss at the level of injury.^10^,^11^ The lateral corticospinal tract carries motor neurons, so damage to the medial portions of the lateral corticospinal tract causes upper extremity weakness.^10^,^11^

CCS occurs in 9% of traumatic spinal cord injuries and is most commonly caused by hyperextension of the neck, which likely occurred in this patient when she fell backwards down the staircase.^10^,^11^ The risk factors for central cord syndrome include advanced age and cervical spondylosis, a condition that weakens the spinal cord.^10^,^11^ However, this patient was noted to have anterior DISH, a condition that increases the risk for cervical fractures and airway obstruction and may have been contributed to the development of the delayed stridor.^5^,^12^

Management of CCS first involves stabilization of the spine, which was done in the ED by placing a cervical collar on this patient.^10^,^11^ Next, the damaged spinal cord is treated surgically with decompression, alignment, and internal fixation, which was completed by an orthopedic surgeon in this case.^10^,^11^ Following surgery, patients are typically transferred to a rehabilitation center for assistance in regaining motor and sensory functions, as was seen in this case as well.^10^,^11^

The key takeaway from this case is that there should be a low level of suspicion for impending upper airway compromise in elderly patients presenting to the ED with a cervical spine injury. It is particularly important to carefully monitor the patients who also have a medical history that places them at an increased risk for airway complications such as obesity, obstructive sleep apnea, male gender, lung diseases, dysphagia, or certain anatomical characteristics like decreased mouth opening or short neck. If a patient develops respiratory distress following a cervical spine injury, then repositioning of the patient’s airway and a breathing treatment may initially be attempted to alleviate the respiratory distress. However, it is important to be prepared to manage a difficult airway with intubation or a surgical airway.

## Supplementary Information




















