# Supplementary figures and images for: Case Report of the Unusual Presentation of Stridor in an Elderly Patient Following a Cervical Fracture
Source: J Educ Teach Emerg Med. 2020 Jan 15;5(1):V15–9. doi: 10.21980/J8V926 (PMC10332537; doi:10.21980/J8V926)

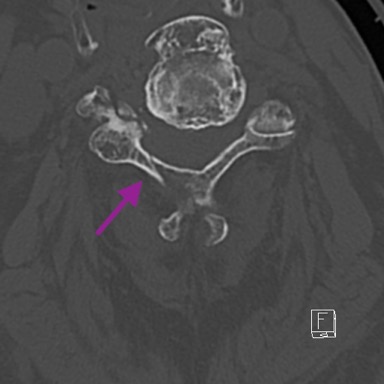

Supplement: Supplementary file 1 [file jetem-5-1-v15-supp1.jpg]

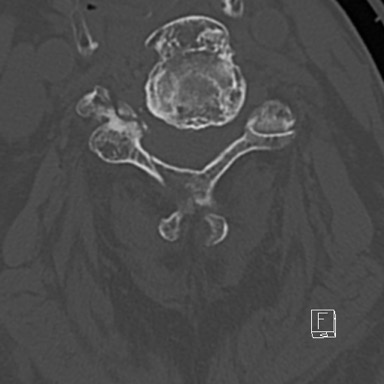

Supplement: Supplementary file 2 [file jetem-5-1-v15-supp2.jpg]

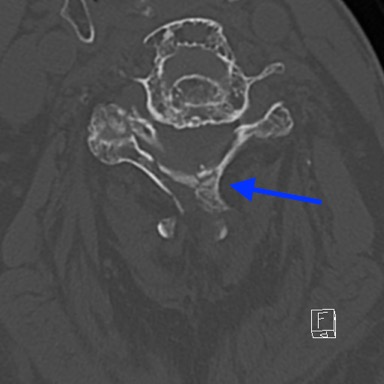

Supplement: Supplementary file 3 [file jetem-5-1-v15-supp3.jpg]

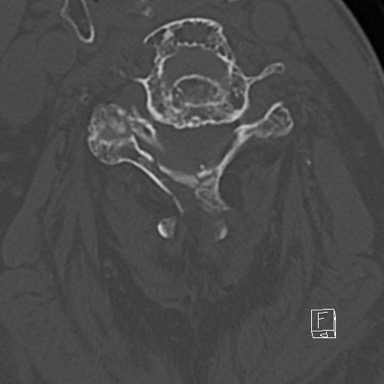

Supplement: Supplementary file 4 [file jetem-5-1-v15-supp4.jpg]

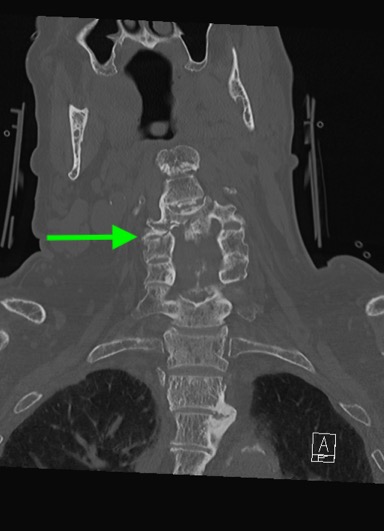

Supplement: Supplementary file 5 [file jetem-5-1-v15-supp5.jpg]

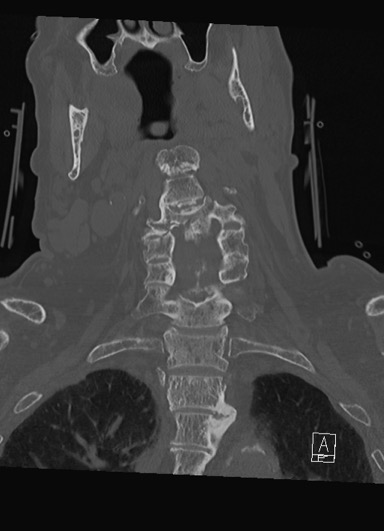

Supplement: Supplementary file 6 [file jetem-5-1-v15-supp6.jpg]

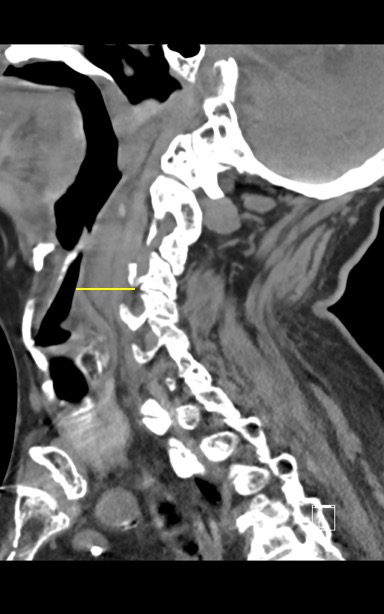

Supplement: Supplementary file 7 [file jetem-5-1-v15-supp7.jpg]

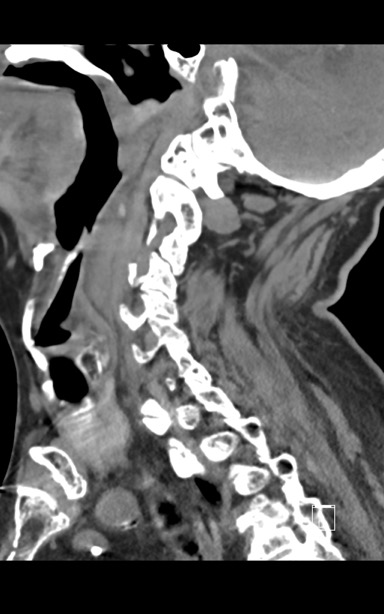

Supplement: Supplementary file 8 [file jetem-5-1-v15-supp8.jpg]

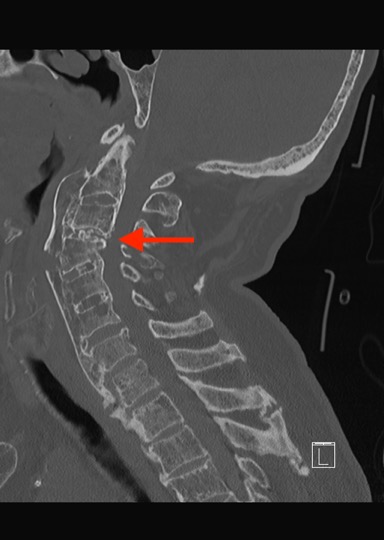

Supplement: Supplementary file 9 [file jetem-5-1-v15-supp9.jpg]

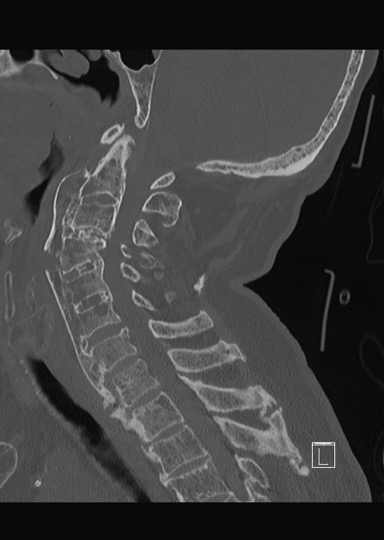

Supplement: Supplementary file 10 [file jetem-5-1-v15-supp10.jpg]
